# Ginsenosides: an immunomodulator for the treatment of colorectal cancer

**DOI:** 10.3389/fphar.2024.1408993

**Published:** 2024-06-12

**Authors:** Jianan Qian, Yanyu Jiang, Hongyi Hu

**Affiliations:** ^1^ Department of Gastroenterology, Longhua Hospital, Shanghai University of Traditional Chinese Medicine, Shanghai, China; ^2^ Institute of Digestive Diseases, Longhua Hospital, Shanghai University of Traditional Chinese Medicine, Shanghai, China; ^3^ Cancer Institute, Longhua Hospital, Shanghai University of Traditional Chinese Medicine, Shanghai, China

**Keywords:** colorectal cancer, ginsenosides, immunomodulation, panax ginseng, traditional Chinese medicine, tumor microenvironment

## Abstract

Ginsenosides, the primary bioactive ingredients derived from the root of Panax ginseng, are eagerly in demand for tumor patients as a complementary and alternative drug. Ginsenosides have increasingly become a “hot topic” in recent years due to their multifunctional role in treating colorectal cancer (CRC) and regulating tumor microenvironment (TME). Emerging experimental research on ginsenosides in the treatment and immune regulation of CRC has been published, while no review sums up its specific role in the CRC microenvironment. Therefore, this paper systematically introduces how ginsenosides affect the TME, specifically by enhancing immune response, inhibiting the activation of stromal cells, and altering the hallmarks of CRC cells. In addition, we discuss their impact on the physicochemical properties of the tumor microenvironment. Furthermore, we discuss the application of ginsenosides in clinical treatment as their efficacy in enhancing tumor patient immunity and prolonging survival. The future perspectives of ginsenoside as a complementary and alternative drug of CRC are also provided. This review hopes to open up a new horizon for the cancer treatment of Traditional Chinese Medicine monomers.

## 1 Introduction

Colorectal cancer (CRC) is the fourth most lethal cancer worldwide, causing nearly 900,000 deaths annually. In addition to aging populations and dietary habits in high-income countries, adverse risk factors such as obesity, lack of physical exercise, and smoking also increase the illness risk (Patel et al., 2022). The tumor microenvironment (TME) is a critical factor in the tumor progression (Dekker et al., 2019). In recent years, the TME has become one of the hotspots of tumor molecular biology research, helping researchers to understand the role of a complex ecosystem composed of tumor cells, immune cells, cancer-associated fibroblasts (CAF), endothelial cells, mural cells, additional tissue-resident cells, and the dynamic, vascularized extracellular matrix in which these cells are embedded.

Increasing evidence suggests that the TME, which supports the tumor, is an essential ecosystem for malignant cells to obtain sufficient oxygen and nutrient supply to meet their high metabolic demands, ultimately leading to cancer (Visser and Joyce, 2023). The correlation between the degree of immune cell enrichment, composition, and functional differences in the TME and the occurrence and development of CRC has been strongly confirmed (Chen et al., 2021; Mei et al., 2021; Liu et al., 2022; Zhong et al., 2022). Therefore, blocking the transition to an immune-suppressive TME has become a promising CRC treatment strategy. In 1979, Lord *et al*. (Lord et al., 1979) proposed the “seed and soil” theory of the TME, likening tumor cells to “seeds” and other components that maintain the growth of tumor cells as “soil” (including immune cells, glial cells, and extracellular matrix, etc.). Since then, many researchers have researched and provided additional data based on this classic concept. They proposed that the intrinsic characteristics of tumor cells, including genetic changes, epigenetic changes, metabolic reprogramming, and signal release, are key determinants of the tumor shaping its microenvironment, and therapeutic interventions may affect the TME and be affected by the TME and systemic changes in the patient (Xiao and Yu, 2021).

Although many drugs are available for regulating TME, the primary strategy is to enhance the anti-tumor ability of T cells by inhibiting immune checkpoints. They are not enough to reverse the progression of CRC. Currently, the latest guidelines (Mi et al., 2023) stipulate that pembrolizumab, programmed cell death protein 1 (PD-1) inhibitor, is applicable as a first-line treatment for patients with unresectable or metastatic high microsatellite instability (MSI-H) or mismatch repair gene defect type (dMMR) CRC with all wild-type KRAS, NRAS, and BRAF genes. MSI-H/dMMR advanced second-line and above CRC patients accept immunotherapeutic drugs, including PD-1/PD-Ligand 1 (PD-L1) inhibitors, such as nivolumab (Lenz et al., 2022), enfortumab (Li et al., 2021). The same kind of PD-1/PD-L1 inhibitors also include tislelizumab and carrelizumab (Yi et al., 2022). Other drugs include cytotoxic T lymphocyte-associated protein 4 (CTLA4) inhibitors (Paillon and Hivroz, 2023), CSF1R inhibitors (Wen et al., 2023), C-C motif chemokine ligand 2 (CCL2) or C-C motif chemokine receptor 2 (CCR2) inhibitors (Goenka et al., 2023), CD47/signal regulatory protein *α*(SIRP*α*) complex antagonists (Li et al., 2023), co-stimulatory molecules such as CD40 (Sadeghlar et al., 2021), and inhibitors of the protein PI3K*γ* (Lee et al., 2020). Candonilimab (Cervantes et al., 2023) (PD-1/CTLA-4 bispecific antibody) was launched this year.

Ginseng is considered a precious herb used to treat various diseases for thousands of years, and its use as a dietary supplement is gradually increasing in North America, Europe, and other countries. Yadav et al. (2024) The pharmacological properties of ginseng are attributed to its various active ingredients (Kang et al., 2021), among which ginsenosides are the main substances (Chen et al., 2022). Numerous studies have shown that the ginsenosides affect various metabolic pathways in the body through various physiological activities, mainly inhibiting tumor cell growth and invasion (Chung and Park, 2016; Jin et al., 2021), inhibiting tumor microvascular formation (Zhu et al., 2021), promoting tumor cell apoptosis (Yin et al., 2021), etc. Currently, the ginsenosides are widely used in the prevention and treatment of cancers, such as intestinal cancer (Xing et al., 2000; Mancuso and Santangelo, 2017; Sun et al., 2022; Chen et al., 2023; Jiang et al., 2023), lung cancer (Peng et al., 2020), breast cancer (Zhu et al., 2023).

Ginsenosides have gradually attracted the attention of researchers, and their role in reversing immune-suppressive TME has received special attention (Sekar et al., 2022; Zhao et al., 2022). Increasing evidence shows that ginsenosides benefit the intestinal ecological environment of normal people and CRC patients and animals (Hou et al., 2022; El-Banna et al., 2022; Lei et al., 2022). Clinical data show that CRC patients taking ginsenosides induce tumor cell cycle blockage and cell apoptosis (Li et al., 2020), reduce cancer-induced collective fatigue (Kim et al., 2020), and inhibit tumor metastasis (Yun et al., 2010). Ginsenosides are a natural drug component with complex components, multiple targets, multiple pathways, and few side effects.

This article encompasses the structure of ginsenosides and their impact on the colorectal TME. More importantly, we highlight research on the immunomodulatory effects of ginsenosides, focusing on their role and mechanisms in promoting immune responses in the TME. By integrating the latest clinical research evidence, this review comprehensively evaluates the potential and prospects of ginsenosides in treating CRC, providing a scientific basis and reference for future clinical applications.

## 2 Ginsenosides are the main active ingredients of ginseng

### 2.1 Structure and classification of ginsenosides

**FIGURE 1:**
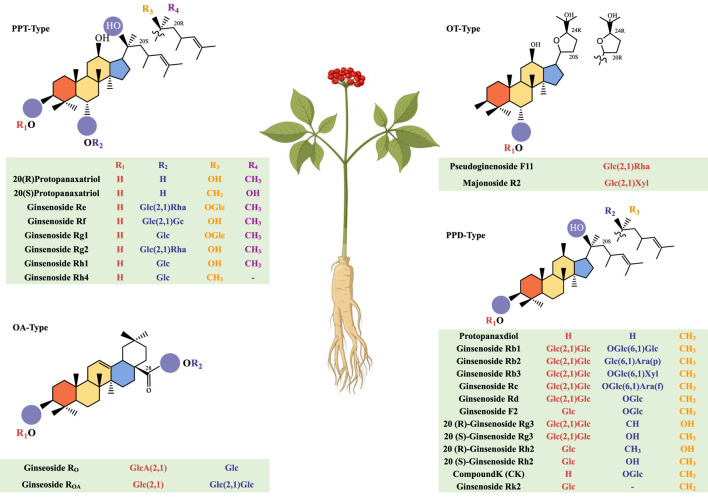
Morphology of the ginseng plant and the chemical structure of ginsenosides.

Ginsenosides are one of the classic triterpenoid compounds composed of aglycones and sugar ligands. They are divided into four types based on the structural features of the core skeleton: protopanaxadiol (PPD), protopanaxatriol (PPT), oleanolic acid (OA), and ocotillol (OT) (Figure 1). Over a hundred natural ginsenoside monomers have been isolated and purified using scientific techniques (Yin et al., 2021; Li et al., 2023). Among them, PPD and PPT are the main active ingredients. Their chemical structures are very similar, and both are tetracyclic triterpene saponins composed of 17 carbons. Different types of PPD-type ginsenosides and PPT-type ginsenosides have different types according to the position and number of hydroxyl groups. PPD-type ginsenosides with glycosidic bonds at C-3 and/or C-20 include protopanaxdiol, ginsenoside Rb1, Rb2, Rb3, Rc, Rd, F2, 20(R)-ginsenoside Rg3, 20(S)-ginsenoside Rg3, 20(R)-ginsenoside Rh2, 20(S)-ginsenoside Rh2, ginsenoside compound K (CK) and Rk2. PPT-type ginsenosides characterized by glycosidic bonds at C-6 and/or C-20 include ginsenoside Re, Rf, Rg1, Rg2, Rh1, and Rh4. OA-type ginsenosides mainly include ginsenoside Ro and others. OT-type ginsenosides mainly include Pseudoginenoside F11 and others.

### 2.2 Ginsenoside content variation and the biosynthesis pathways

In recent years, new biosynthesis of ginsenosides has been directly related to the activity of squalene monooxygenase. Biosynthesis starts from the mevalonic acid pathway and the methylerythritol-4-phosphate/deoxyxylulose-5-phosphate pathway to produce farnesyl diphosphate, which is converted into 2,3-epoxy squalene through the action of squalene synthase and squalene epoxidase (Seki et al., 2015). The OA-type saponin precursor OA is carboxylated to form via *β*-Amyrin synthase and OA synthase (Kim et al., 2015). The PPD-type saponin precursor PPD and PPT-type saponin precursor PPT (Hou et al., 2021) are produced separately by hydroxylation via dammarenediol synthase, PPD synthase, or PPT synthase. The OT-type saponin precursor OT (Yang et al., 2023) is produced by catalysis via PgOSC11. After the basic skeletons of PPD, PPT, OA, and OT are synthesized, the subsequent structures of ginsenoside biosynthesis are completed by various uridine diphosphate-dependent glycosyltransferases catalyzing glycosylation modification or acyltransferases catalyzing acylation modification (Shi et al., 2023). Recently, Hu *et al*. (Hu et al., 2023) found that using supercritical CO_2_ to extract total ginsenosides from ginseng leaves better retains the activity of ginsenosides.

The content of ginsenosides in ginseng varies according to the part. Some scholars have evaluated the correlation of ginseng tissue samples. The results show that the content of ginsenosides is similar among the same ginseng, and the content of ginsenosides in ginseng leaves, leaf stalks, and stems shows a decreasing trend, with the roots containing a larger amount of ginsenosides (Xu et al., 2017).

The type of ginsenosides does not vary much with different varieties of ginseng, mainly depending on the part of the ginseng. Recently, Jin *et al*. (Jin et al., 2022) established a ginsenoside mass spectrometry database, detecting 174 kinds of ginsenoside monomers or isomers (69 PPD-type ginsenosides, 63 PPT-type ginsenosides, 22 OA-type ginsenosides, 11 OT-type ginsenosides, and seven other types of ginsenosides). There are large differences in ginsenosides between different ginseng tissues, leaves, or roots. PPT-type ginsenosides have a higher abundance in leaves, PPD-type ginsenosides have a higher content in roots than in leaves, and OA and OT-type ginsenosides are evenly distributed (Luo et al., 2023).

## 3 Ginsenosides regulate immune response in the colorectal tumor microenvironment

### 3.1 Lymphocytes

Most CRC patients have an immune dysfunction state, characterized by a decrease in CD4^+^T cells and a decrease in the ratio of CD4^+^T/CD8^+^T cells (Robins et al., 1991; Li et al., 2020). CD4^+^T cells drive qualitative changes in anti-cancer immune responses (DiToro and Basu, 2021). CD8^+^T cells cause tumor cell death by releasing granzymes and perforins or through fatty acid synthase ligand (FASL)-fatty acid synthase (Fas)-mediated cell apoptosis. High infiltration of CD8^+^T cells indicates a better prognosis and a more satisfactory immune therapy response (Shang et al., 2022). The T-helper1 (Th1) subtype of CD4^+^T cells assists cytotoxic T cells and B cells in producing Interferon-*γ* (IFN-*γ*) and Tumor necrosis factor-*α* (TNF-α) to directly kill cancer cells (Woznicki et al., 2020). In contrast, the Th2 subtype secretes anti-inflammatory mediators, which have a pro-tumor effect (Hombach et al., 2020). In the tumor tissues and peripheral blood of humans and mice, there is an increase in the expression of the Regulatory T cell (Treg) subgroup (TSLPR^+^ Tregs) of the thymic stromal lymphopoietin (TSLP) receptor, which does not exist in the peripheral blood of adjacent normal colon tissues and healthy controls (Obata-Ninomiya et al., 2022). Tregs are a highly immunosuppressive subgroup, serving as the “gatekeeper” of the steady-state CRC immune microenvironment (Obata-Ninomiya et al., 2022). In the tertiary lymphoid structures within the tumor, B cells promote T cell activation through antigen presentation. B cells also support tumor growth, specifically by secreting pro-angiogenic mediators, immune complexes, and complement activation to promote inflammation and immune suppression (Xia et al., 2023). Invariant natural killer T-cells are enriched in CRC tumor lesions.

**FIGURE 2:**
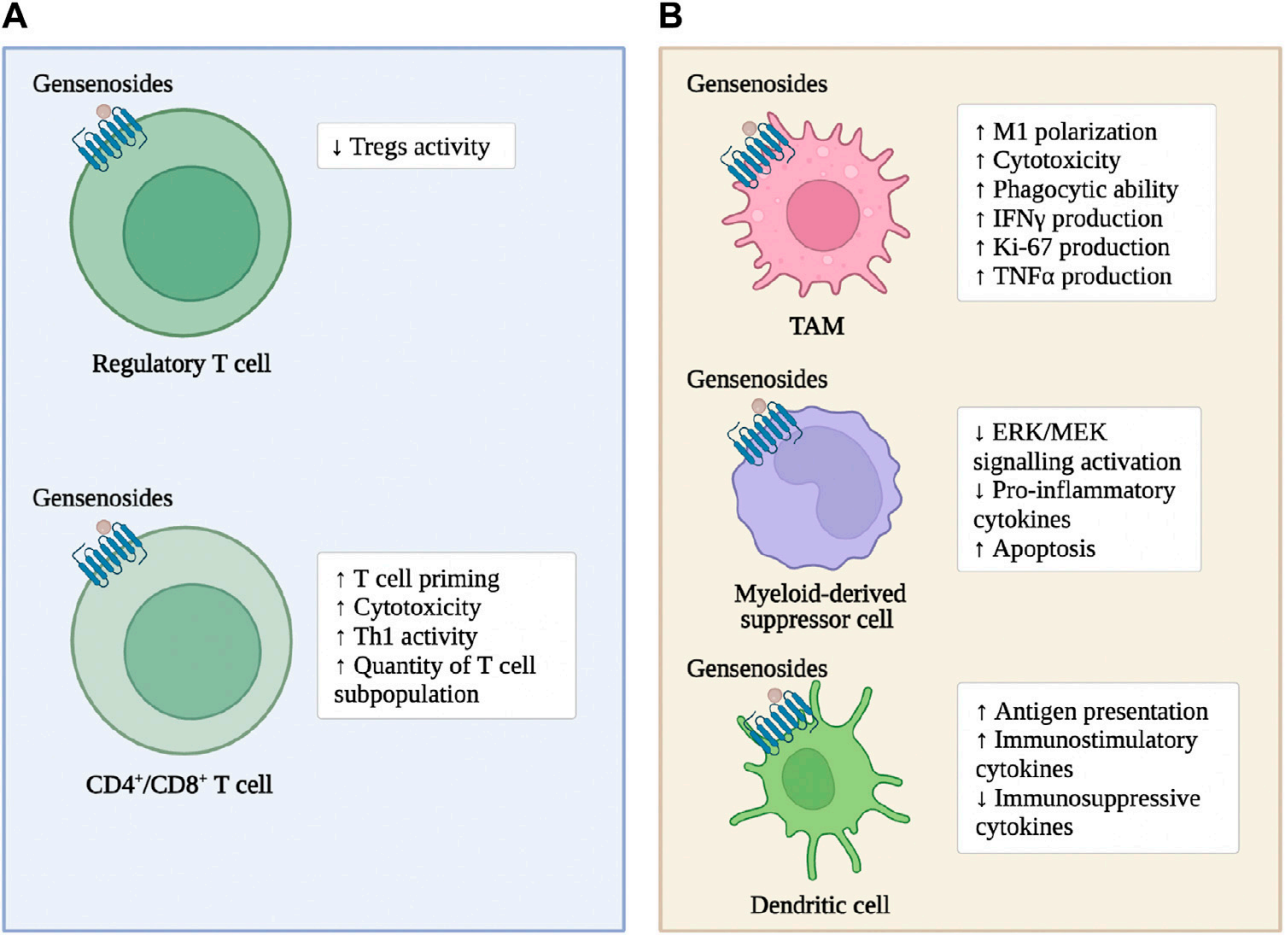
Ginsenosides modulate immune cells in CRC. **(A)**: the immune cells of the Lymphocytes. **(B)**: the immune cells of the myeloid systems.

As previously mentioned, ginsenosides enhance the immune response of adaptive immune cells within CRC tumor tissue and enhance the cytotoxic effect on tumor cells (Figure 2). In the subcutaneous transplantation tumor model of MC38 in mice, ginsenoside Rh2 enhances the anti-tumor effect of anti-PD-L1 antibodies. The mechanism is that the combined treatment increases the expression of C-X-C motif ligand 10 (CXCL10), thereby promoting the infiltration and activation of CD8^+^T cells within the tumor (Huang et al., 2023). Ginsenoside Rh2, Rg3, and CK block the interaction between PD-1 and its ligand PD-L1, enhancing the activity of cytotoxic T cells (Yim et al., 2020). In the subcutaneous transplantation tumor model of MC38 in mice expressing humanized PD-1/PD-L1, red ginseng containing a large amount of ginsenoside Rh2, Rg3, and CK significantly inhibit tumor growth, increases the infiltration of CD8^+^T cells into the tumor, and enhance the production of granzyme b (Lee et al., 2023). Ginsenoside Rb1 and Rc reduce the number and size of intestinal adenomas in mouse models of intestinal adenomas. The mechanism is to inhibit the activation of Tregs induced by hypoxia-inducible factor-1*α* (HIF-1*α*) in tumor tissues (Xu et al., 2018). Ginsenoside Re and ginsenoside Rd significantly promote the adaptive immune response in the CRC microenvironment. The mechanism is to increase the Th1 activity, inhibit the differentiation of T helper cell 17 (Th17) and regulate the balance of Th17/Treg (Wang et al., 2021). Ginsenoside Rg1 improves the structure of the microbial community in the intestine and restore the intestinal homeostasis. The mechanism is to downregulate the proportion of Th17 cells (Zhang et al., 2022).

### 3.2 Myeloid cells

Myeloid immune cells include macrophages, neutrophils, platelets, and others. Tumor-associated macrophages (TAM) are recruited from peripheral circulating monocytes to the tumor microenvironment, where they differentiate into pro-inflammatory, antigen-presenting, and anti-tumor M1 phenotypes or immunosuppressive M2 phenotypes. The M2 phenotype also promotes tumorigenesis by promoting angiogenesis, metastasis, and treatment resistance (Wang et al., 2021). Systemic accumulation of neutrophils contributes to immune suppression and extracellular matrix (ECM) remodeling in distant organs (He et al., 2022). Neutrophils promote the formation of pre-metastatic niches by forming sticky net-like structures called neutrophil extracellular traps that trap circulating cancer cells in distant inflamed organs (Khan et al., 2021). Monocytes also differentiate into tumor-supporting TAMs to promote an immunosuppressive state in the TME (Lam et al., 2021). Dendritic cells (DC) play a central role in regulating the balance between CD8^+^ T cell immunity and tumor antigen tolerance by integrating information from the TME and transmitting it to T cells and other immune cells, forming an anti-tumor immune response (Fu and Jiang, 2018). Mast cells exert pro-tumor and anti-tumor activities depending on environmental stimuli (Sakita et al., 2022). Eosinophils have the ability to directly kill CRC cells by releasing cytotoxic molecules (Grisaru-Tal et al., 2022). Myeloid-derived suppressor cells (MDSC) have potent immunosuppressive abilities, inhibiting T cells, natural killer (NK) cells, B cells, and DC cells through paracrine secretion and cell-cell contact (Kumar et al., 2016). Platelets are activated and aggregated by circulating tumor cells (CTC), promoting CRC metastasis and related immune escape by protecting CTCs from physical stress and immune attack (Liang et al., 2015; Kanikarla-Marie et al., 2017; Pereira-Veiga et al., 2022).

As previously mentioned, ginsenosides enhance the tumor-suppressing characteristics of innate and adaptive immune cells in the CRC microenvironment to induce tumor cell apoptosis and enhance the anti-tumor activity of chemotherapeutic drugs (Figure 2). Ginsenoside Rg3 increases the number of leukocytes in colon cancer patients and promotes the phagocytic ability of macrophages (Zhu and Gao, 2021). Cyclophosphamide is still widely used as an anti-tumor agent and immunosuppressant in clinical practice (Taniura et al., 2020). Ginsenoside Rh2 enhance cyclophosphamide’s anti-tumor activity by reducing micronuclei formation in polychromatic erythrocytes and Deoxyribo Nucleic Acid (DNA) strand breaks in leukocytes (Qi et al., 2019). In MC38 cells co-cultured with TAMs, ginsenosides inhibit the invasion and migration of colon cancer cells, and the mechanism is that Rh2 inhibit the polarization of TAMs to M2 macrophages (Liu et al., 2023). In addition, the abnormal immune response-mediated inflammatory response is a key factor in promoting the carcinogenesis of ulcerative colitis (UC) to CRC, and slowing down the inflammatory process prevents the carcinogenesis of UC (Yang et al., 2013; Li et al., 2021). Ginsenoside Rk2 reduce the secretion of pro-inflammatory cytokines, such as Interleukin-1*β* (IL-1β), Interleukin-6 (IL-6), Interleukin-10 (IL-10), TNF-α, in the vitro UC model established by co-culturing Caco-2 cell clones with THP-1 cells in a concentration-dependent manner, and slow down the inflammatory process by inhibiting the activation of the extracellular signal-regulated kinase (ERK)/mitogen-activated protein kinase (MEK) pathway (Huang et al., 2022).

## 4 Ginsenosides modulate matrix cells and matrix components in the colorectal tumor microenvironment

### 4.1 Cancer-associated fibroblasts

The origin of CAF is still debated among researchers. Kamali *et al*. recently proposed that CAFs originate from bone marrow-derived mesenchymal cells, specifically triggered by C-X-C motif chemokine ligand 12 (CXCL12) signaling and transforming growth factor *β* (TGF-*β*) (Kamali Zonouzi et al., 2022). In the early stages of CRC formation, there is an increase in the proliferation of connective tissue in the colon, specifically characterized by a large proliferation of colorectal cancer fibroblasts, with the highest proportion being leprosy interstitial cells (Kobayashi et al., 2022). Studies have found that colon cancer cells induce tumor formation by producing 12(S)-HETE acting on CAFs (Stadler et al., 2017). Different tissues in the colon have different fibroblast lineages, leading to different subgroups of CAFs with different cellular states or functions (Costa et al., 2018). As the tumor progresses further, the composition and function of CAFs change, specifically through the production of large amounts of fibrosis, chemotactic factors, and different factors (such as fibroblast growth factors, FGFs) to form a microenvironment that supports CRC (Visser and Joyce, 2023). CAFs express different factors, including *α*-smooth muscle actin (*α*SMA), vimentin, WNT_2_, Fibroblast activation protein (FAP), and Gremlin1 (GREM1), among which WNT_2_ (Aizawa et al., 2019) and GREM1 (Karagiannis et al., 2013) mainly promote tumor metastasis. Activin A secreted by CAFs plays a major role in TGF-*β*-induced pre-metastatic changes in epithelial cells (David and Massagué, 2018). It can be said that CAFs play an important role in both the formation and metastasis of CRC.

**FIGURE 3:**
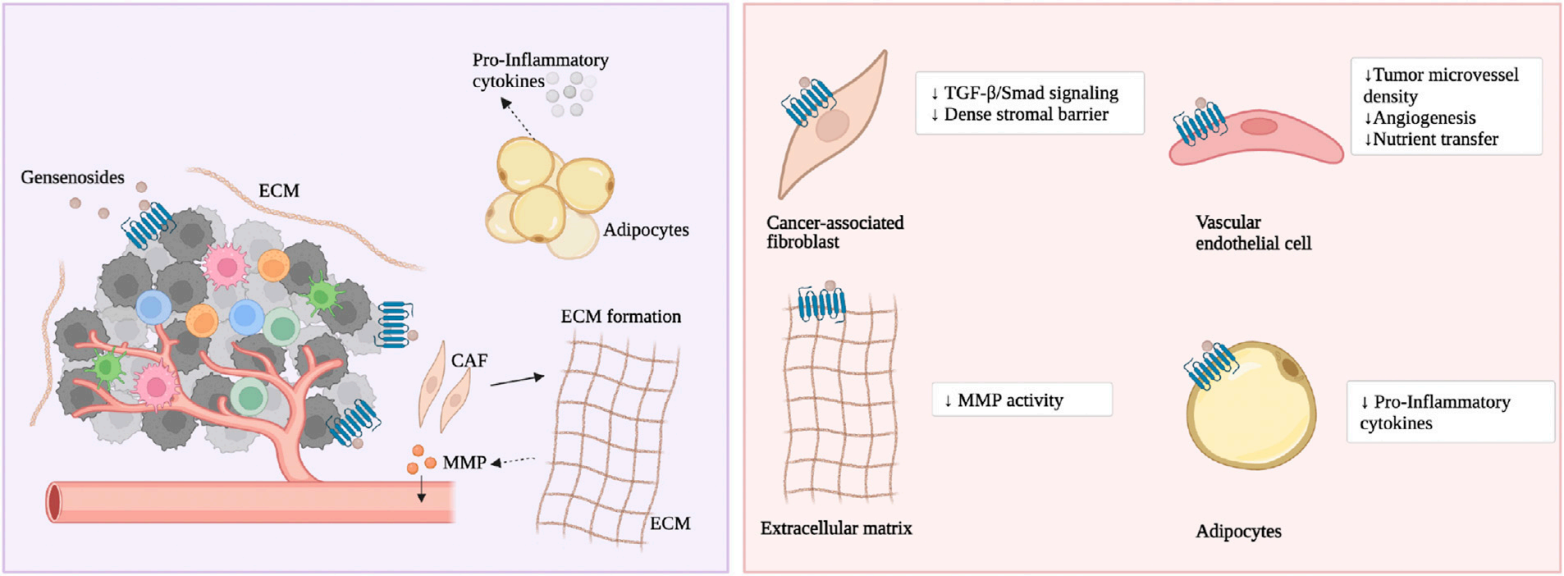
The main molecular mechanism of ginsenosides on the matrix cells and matrix components. Ginsenosides treat CRC by modulating various stromal components.

As previously mentioned, ginsenoside Rg3 and Rd prevent CRC formation prophylactically. The mechanism is that ginsenoside Rg3 and Rd inhibit CRC cells from secreting TGF-*β*, and through the TGF-*β*/Smad signaling pathway, they reverse activated CAFs to a resting state, thereby weakening the dense interstitial barrier within the tumor (Huang et al., 2017), depicted in Figure 3. Rg3 and Rd also promote the recovery of physiological functions of the intestinal epithelium. The mechanism is that Rg3 and Rd downregulate oncogenic signaling molecules iNOS, signal transducer and activator of transcription 3 (STAT3), phosphorylated STAT3, sarcoma gene (Src) and phosphorylated Src, promote the secretion function of goblet cells and Paneth cell clusters, and restore the expression of E-cadherin and N-Cadherin (Huang et al., 2017). Currently, there are not many studies on the effect of ginsenosides on CAFs in CRC. Existing research focuses on ginsenosides promoting the expression of glutathione to alleviate fibroblast proliferation caused by smoke stimulation. Ginsenoside Rg1, Rb1, and Rg3 downregulate the mRNA and protein levels of multidrug resistance-associated protein 1 (MRP1), basic fibroblast growth factor (bFGF), and fibroblast growth factor receptor 1 (FGFR1), and reduce the protein expression of glutathione S-transferase *π* (GST-*π*) (Weiqin et al., 2011; Jin et al., 2017). Rg3 also enhances the vitality of matrix cells and weakens cancer cell migration. The mechanism is to promote the cell cycle transition from the G0/G1 phase to the S phase and inhibit the cancer cell-related fibroblast-like phenotype (Peng et al., 2017).

### 4.2 Cancer-associated adipocytes

Cancer-associated adipocytes (CAA) influence cancer cells and host cells in the TME by releasing metabolites, growth factors, and inflammatory adipokines (Iacono et al., 2022). There is an active exchange of metabolites between CAAs and cancer cells; specifically, CAAs release metabolites such as adenosine triphosphate (ATP), lactate, pyruvate, and glutamine into the colorectal cancer microenvironment, increasing the oxidation of fatty acids in cancer cells (Wu et al., 2021). CAAs promote metabolic reprogramming and chemoresistance in colon cancer cells. CAAs release adipokines and metabolic factors to regulate the immune response of anti-tumor immune cells (Harmon et al., 2019; Grigoras and Amalinei, 2023). CAAs contribute to the formation of a low-grade chronic inflammatory environment conducive to tumorigenesis, the mechanism being that CAAs influence the TME by releasing pro-inflammatory factors IL-1β, IL-6, IL-8, and TNF-α (Grigoras and Amalinei, 2023). In addition, CAAs are frequently located at the front of invasive colorectal cancer, exhibiting a fibroblast-like phenotype, and establish a bidirectional molecular dialogue with colorectal tumor cells, leading to functional changes in normal cells and promoting CRC invasion (Munteanu et al., 2020).

As previously mentioned, ginsenoside F2 reduce the lipid levels accumulated in the 3T3-L1 cell line during adipogenesis and inhibits the growth of breast cancer cells (Siraj et al., 2015). However, its effect on CRC has not been reported. Ginsenoside Rb2 improve TNF-*α*-induced apoptosis in 3T3-L1 adipocytes, the mechanism being that Rb2 reduces the phosphorylation levels of p65 and I*κ*B*α* in the nuclear factor *κ*B (NF-*κ*B) pathway both *in vitro* and *in vivo* to inhibit adipocyte apoptosis (Lin et al., 2020). Ginsenoside Rg1 promotes the neural differentiation of mouse adipose stem cells through the miRNA-124 signaling pathway (Dong et al., 2017). Ginsenoside Rh1 (Gu et al., 2013), Rh2 (Hwang et al., 2007), Rb1 (Wang et al., 2017; Cai and Chen, 2021), Rg3 (Hwang et al., 2009), and F2 (Zhou et al., 2021) inhibit adipocyte differentiation while also suppressing the overexpression of adipokines (peroxisome proliferator-activated receptor *γ*, PPAR*γ*) and inflammatory factors (such as TNF-α). Ginsenoside CK and Rg3 inhibit early adipocyte formation through the Adenosine 5’-monophosphate (AMP)-activated protein kinase (AMPK), mitogen-activated protein kinase (MAPK), and perine-threonine kinase (AKT) signaling pathways (Oh and Chun, 2022; Jue-Yao et al., 2023). Ginsenoside Rb2 reduces fat accumulation through an AKT-dependent mechanism (Dai et al., 2018). In addition, CRC cell mitochondria often have functional disorders (Ohshima et al., 2022). Ginsenoside Rd improves mitochondrial biogenesis function, the mechanism being that Rd promotes the phosphorylation of TANK-binding kinase 1 (TBK1) and AMPK in adipocytes through the WNT5A/Ca^2+^ signaling pathway, promoting the expression of lipopolysaccharide-induced membrane proteins (Wan et al., 2023).

### 4.3 Extracellular matrix, neurons and nerve fibers

Increasing evidence suggests that the ECM, neurons, and nerve fibers contribute to the formation of CRC. The ECM is a complex network composed of macromolecules (such as collagen, enzymes, proteoglycans, and glycosaminoglycans) secreted by CRC cells, supporting epithelial/endothelial cells, the underlying matrix, and the cell membrane (Nersisyan et al., 2021). It can be said that the degradation of the ECM and the dynamic physical conditions of the TME affected by it are important pathways for the progression and metastasis of CRC (Andreuzzi et al., 2022; Franchi et al., 2023). There is active paracrine signaling crosstalk between neurons and tumor cells. Neurons stimulate cancer stemness, anti-apoptosis, and proliferative ability by releasing neurotransmitters, neurotrophic proteins, and chemokines (Schonkeren et al., 2021; Zhu et al., 2022). Perineural invasion (PNI) (i.e., local extension of CRC cells along nerves) is observed in CRC, which is closely related to poor prognosis (Liu et al., 2022).

As previously mentioned, ginsenoside CK inhibits the activity of matrix metalloproteinases (MMPs), thereby reducing the degradation of various protein components of the ECM (Park and Yoon, 2012). At present, no studies have found the mechanism by which ginsenosides affect neurons in the TME. The author speculates that in the TME, ginsenosides block cancer cells from communicating with the surrounding ECM and nerve fibers through integrins, thereby inhibiting the progression of CRC.

### 4.4 Vascular endothelial cells

Ginsenosides inhibit the formation of blood vessels in the colorectal TME, reducing the transfer of nutrients and lowering the survival rate of CRC cells. Ginsenoside Rg1, Rb1, and Rg3 reduce the density of microvessels in tumors (Jin et al., 2017). Ginsenoside CK inhibits the formation of blood vessels in the colon (Park and Yoon, 2012). Ginsenoside Rg3 slows CRC’s new blood vessel formation rate (Hong et al., 2020), regulate the TME, inhibits CRC cells’ growth, proliferation, and migration, and promotes cell apoptosis (Jian et al., 2016; Xu et al., 2023). Clinical studies have confirmed that the combined use of Rg3 and chemotherapy regulates the level of local vascular endothelial growth factor in CRC to enhance the effect of chemotherapy (Zhu and Gao, 2021).

## 5 Ginsenosides change the basic hallmarks of CRC cells

**FIGURE 4:**
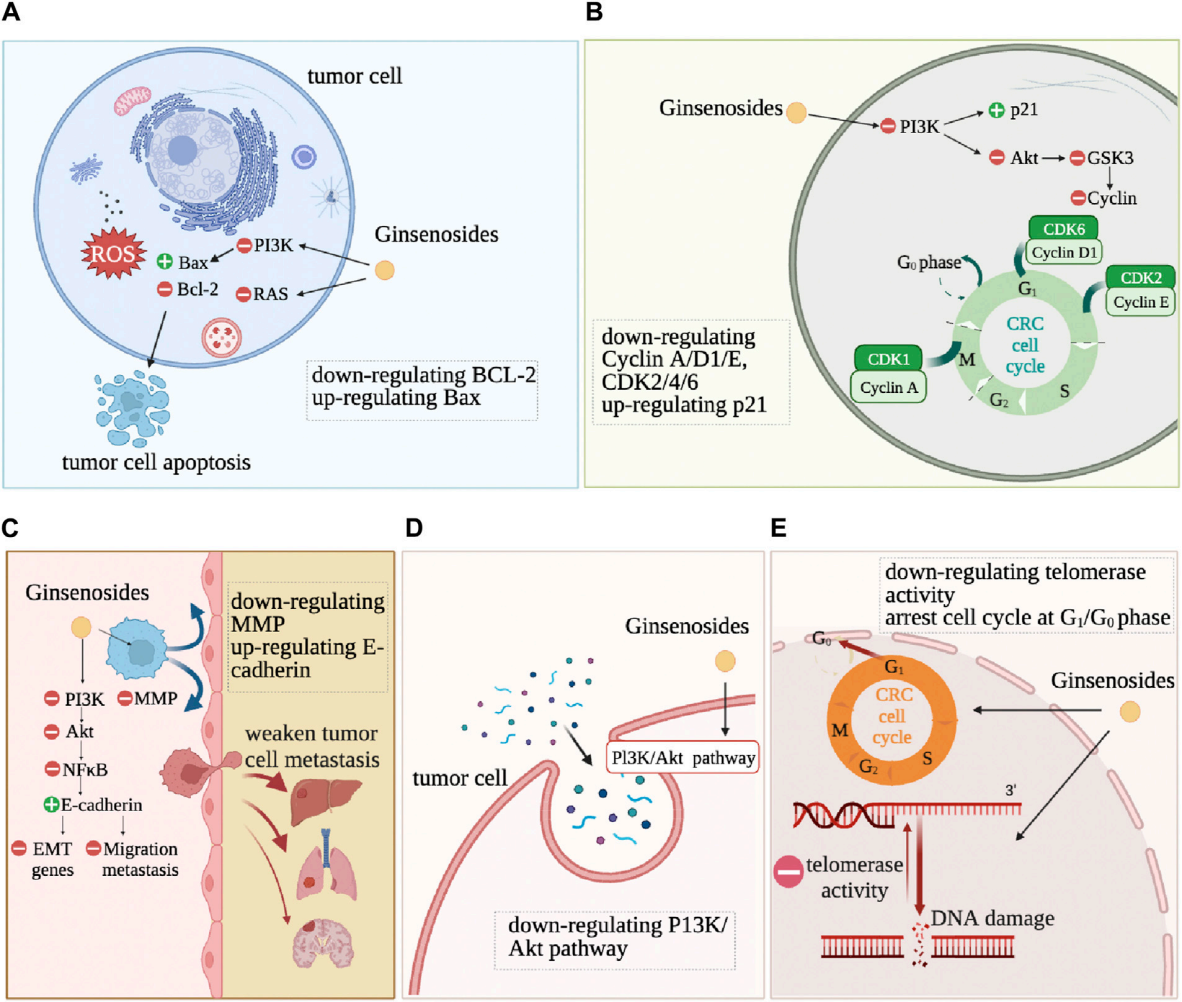
The action mechanism of ginsenosides on tumor cells. DNA damage, cell apoptosis, cell cycle arrest, metastasis inhibition are the most common feature changes after ginsenoside intervention in CRC cells. **(A)**: inducing apoptosis. **(B)**: inducing cell cycle arrest. **(C)**: inhibiting tissue invation and metastasis. **(D)**: regulating autophagy. **(E)**: inducing differentiation.

Ginsenosides alter the characteristics of CRC cells, such as resistance to cell death, continuous proliferation, and drug resistance, as shown in Figure 4. Anti-cell death is one of the basic characteristics of tumor cells. Apoptosis refers to programmed cell death that occurs after irreversible DNA damage (Roos et al., 2016). Ginsenosides induce apoptosis and autophagy by regulating various proteins and molecular pathways. Ginsenoside Rd downregulates the expression of lncRNA membrane-associated guanylate kinase inverted one intronic transcript 1 (MAGI1-IT1), increases the proportion of CRC cells in the G0/G1 phase, reduces the proportion in the S phase, reduces the protein expression levels of CyclinD1, Caspase3, B-cell lymphoma-2 (Bcl-2), increases the expression levels of p21, cleaved-Caspase3, Bax, and ultimately promotes the apoptosis of SW480 CRC cells (Kou et al., 2023) (Figure 4). Ginsenosides Rd and Re induce apoptosis of CRC cells HCT116 and HT29 by regulating the expression of apoptosis-related genes, such as increasing the protein expression levels of p53, p21, Bax, Caspase3, Caspase8, Caspase9, while significantly inhibiting the differentiation of Th17 cells, promoting the adaptive immune response of the CRC microenvironment by regulating the balance of Th17/Treg (Wang et al., 2021). Ginsenoside Rg3 and 5-FU combined treatment significantly enhance the apoptosis of CRC cells by activating the Apaf1/caspase 9/caspase three pathway. It blocks the cell cycle of CRC cells SW620 and LOVO in the G0/G1 phase by promoting the expression of Cyclin D1, cyclin-dependent kinase 2 (CDK2), and CDK4 (Hong et al., 2020). Ginsenoside Rh2 combined with sodium selenite has a synergistic anti-tumor effect on HCT-116 human CRC cells cultured *in vitro*, inducing G1 phase and S phase block, increasing cell apoptosis rate, increasing Bax/Bcl2 ratio and caspase-3 expression, significantly inducing reactive oxygen species (ROS) production and autophagy (Zhu et al., 2016). It has also been proven that Rh2 directly inhibits the activity of PDZ-binding kinase/T-LAK cell-originated protein kinase (PBK/TOPK), inducing the death of HCT116 cells (Yang et al., 2016). Ginsenoside Rh2 and Rg3 both induce the death of HCT116 and SW480 cells, significantly increase the level of pro-apoptotic regulator Bax by activating NF-*κ*B transcriptional activity, and induce CRC cells to die in the form of cytoplasmic vacuole accumulation by reducing the level of anti-apoptotic regulator Bcl-2 (Li et al., 2011).

Continuous proliferation signals are another fundamental characteristic of tumor cells. Normal colon epithelial cells need to activate mitotic growth signals to transition from a resting state to an active proliferation state. However, due to the dysregulation of self-renewal and differentiation signals in CRC cells, overexpression of cell cycle proteins or non-expression of CDK inhibitors allows cancer cells to maintain an active proliferation state (Schwartz and Shah, 2005). Ginsenosides change the self-sufficient growth signals of CRC cells and inhibit the proliferation and vitality of CRC cells. Ginsenoside CK significantly upregulates the expression of cyclin-dependent kinase inhibitor 1A (CDKN1A), downregulates the expression of CDK6, Cyclin D, and Cyclin E, causing G1 phase arrest in CRC HCT-116 and HT-29 cells (Yao et al., 2018). Ginsenoside CK also significantly inhibits human CRC cell proliferation HCT-116 and SW480 (Wang et al., 2012). Ginsenoside 20(S)-PPD (PD), 20(S)-PPT (PT), and Rh2 reduce the vitality of Caco-2 cells (Popovich and Kitts, 2004). Ginsenoside Rb2 inhibits the growth, adhesion, epithelial-mesenchymal transition (EMT) of CRC cells through the TGF-*β*1/Smad signaling pathway (Dai et al., 2019). Ginsenosides Re and Rd significantly inhibit the growth of CRC cells by upregulating cell cycle protein A to inhibit the G2/M cell cycle and inducing apoptosis by regulating the expression of apoptosis-related genes (Wang et al., 2021).

Ginsenosides inhibit the metastasis and invasion of CRC cells by mediating the Epidermal Growth Factor Receptor (EGFR) signaling pathway, NF-*κ*B signaling pathway, and PI3K/Akt signaling pathway. Ginsenoside Rb2 downregulates EMT-related gene expression through the EGFR/SOX2 signaling axis, inhibiting the migration and invasion of CRC cells (Phi et al., 2018). Li *et al*. found that ginsenoside Rh2 induces the death of CRC cells and inhibits cancer cell migration by activating NF-*κ*B transcriptional activity (Li et al., 2011). Rh2 induces the expression of miR491 to inhibit the metastasis of CRC cells (Wei et al., 2021). Ginsenoside Rg3 and 5-fluorouracil combined treatment of CRC cells enhances the anti-tumor effect of 5-fluorouracil in CRC cells and inhibits tumor invasion and migration through the PI3K/AKT pathway (Sun et al., 2017; Liu et al., 2022). 20(S)-ginsenoside Rh2 also inhibit the expression of IL-6-induced STAT3 and MMPs, including MMP-1, MMP-2, and MMP-9, thereby inhibiting the CRC cell invasion (Han et al., 2016).

Ginsenosides enhance the efficacy of chemotherapy drugs for CRC and reduce the drug resistance and side effects of other drug treatments. Oxaliplatin and 5-Fu are the most commonly used first-line chemotherapy drugs for CRC (Yang et al., 2023; Mi et al., 2023), and ginsenosides reverse chemotherapy resistance in CRC. Ginsenoside Rh2 significantly inhibits the proliferation of oxaliplatin (L-OHP) resistant CRC cells (LoVo/L-OHP) and LoVo cells and induces apoptosis in LoVo cells, significantly reducing the expression of P-gp and Bcl-2, increasing the expression levels of Smad4, Bax, and caspase-3, reversing the drug resistance of LoVo/L-OHP cells to L-OHP (Ma et al., 2019). Ginsenoside Rh2 enhance the cytotoxicity of 5-FU to drug-resistant CRC cells (LoVo/5-FU and HCT-8/5-FU), increase the number of drug-resistant CRC cells in the G0/G1 phase, decrease the number of cells in the S phase, and induce cell apoptosis. Ginsenoside Rh2 treatment inhibits the migration process of drug-resistant CRC cells and the EMT process, and the expression of drug-resistant genes MRP1, multidrug resistance protein 1 (MDR1), Low-density lipoprotein receptor-related protein (LRP), and GST is negatively correlated with ginsenoside Rh2 (Liu et al., 2018). Ginsenosides increase the sensitivity of anti-tumor drugs. It has been found that CK combined with chemotherapy drugs enhance the sensitivity of chemotherapy drugs and reverses drug resistance in tumor cells. CK enhances tumor necrosis factor-related apoptosis-inducing ligand (TRAIL)-induced apoptosis in HCT116 CRC cells and increases the sensitivity of CRC HT-29 cells to the drug tolerance of recombinant TRAIL. Combined use leads to a decrease in the expression of proteins that promote cell survival and an increase in the expression of pro-apoptotic proteins and then induces an increase in the expression of death receptor 5 (DR5) on the cell surface (Chen et al., 2016). Ginsenoside Rp1 inhibits AKT activation and SIRT1 upregulation induced by Actinomycin D. Ginsenoside Rp1 combined with chemotherapy drugs avoid drug resistance and enhance the anti-tumor effect of drugs (Yun et al., 2020). In addition, the standard treatment methods for CRC will cause damage to normal tissue organs while killing cancer cells, such as myocardial cell damage and intestinal flora disorder. Rh2 reduces local pathological remodeling by reducing the transformation of cardiac fibroblasts to myofibroblasts (FMT) and endothelial-mesenchymal transition (EndMT) (Hou et al., 2022). Ginsenoside CK significantly inhibits the growth of CRC in mice and, by significantly upregulating the adhesion bacteria that inhibit the proliferation of human CRC cells, restores the disordered intestinal flora of tumor-bearing mice (Shao et al., 2022).

## 6 Ginsenosides regulate the physicochemical properties of tumor microenvironment

**FIGURE 5:**
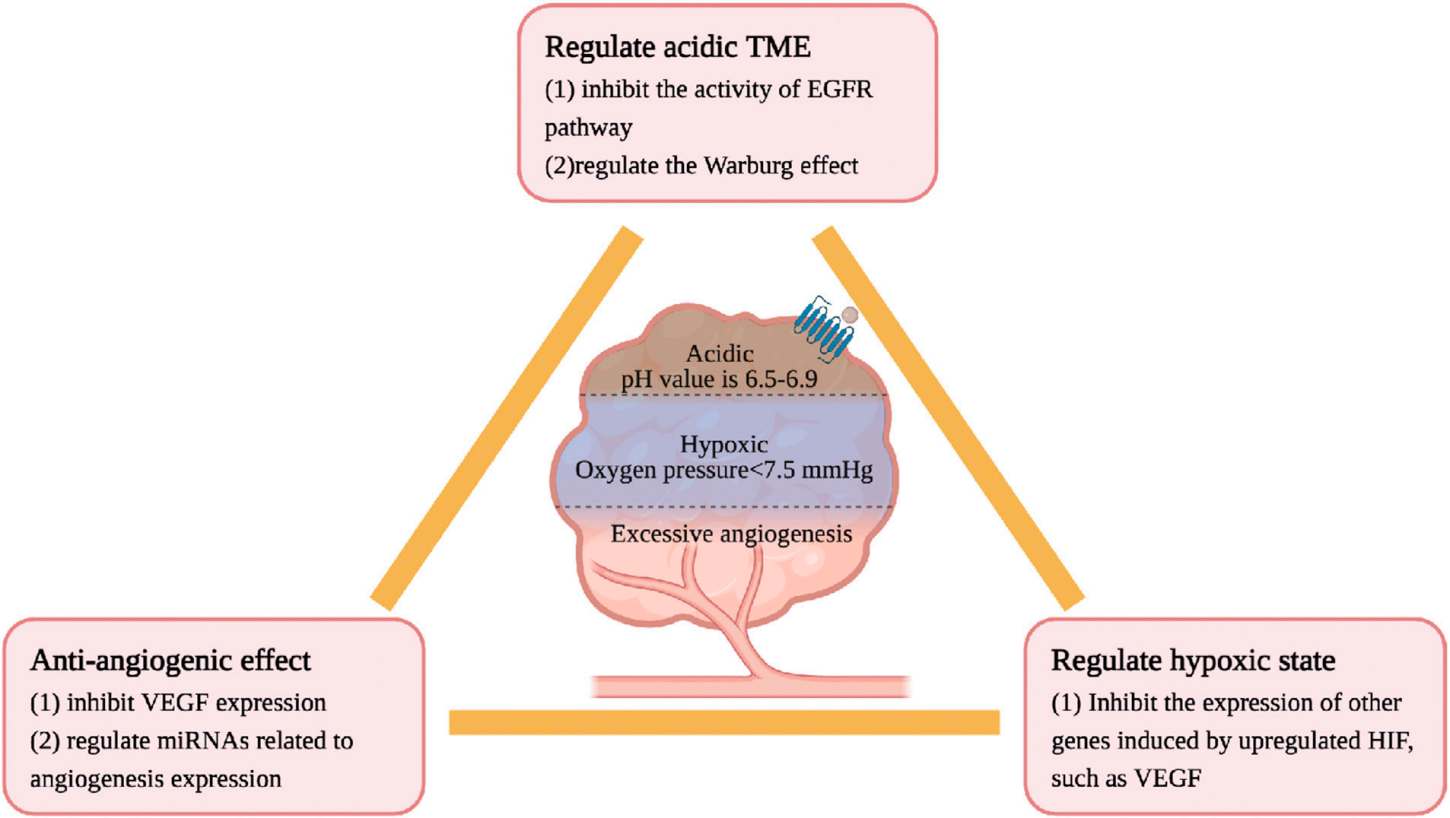
Ginsenosides regulate the physicochemical properties of TME characterized by hypoxia, low pH and excessive angiogenesis.

The physicochemical properties of TME are different from those of the normal internal environment, including hypoxia, low pH, high pressure, and excessive angiogenesis, all of which lead to the accelerated development of CRC. Ginsenosides inhibit the growth of CRC by regulating the physicochemical properties of the TME. The details are as follows, see Figure 5.

CRC cells have a high oxygen consumption and relatively insufficient oxygen supply, often in a state of hypoxia and high permeability of tumor blood vessels. Under normal circumstances, the oxygen partial pressure of human tissues is about 40 mmHg, while the oxygen partial pressure of most TMEs is less than 7.5 mmHg (Casazza et al., 2014). In a low oxygen microenvironment, the HIF level will be upregulated, thereby increasing the expression of other genes such as VEGF, inducing the formation of new blood vessels, and maintaining a stable state of oxygen (Ma et al., 2022). Although no studies have confirmed a direct association between ginsenosides and HIF, current research confirms that ginsenosides have anti-angiogenic effects in intestinal tumors. Rg3 inhibits the expression of vascular endothelial growth factor (VEGF) and prolongs the lifespan of CT26 CRC model mice (Liu et al., 2018). Rh2 regulates the expression of miRNAs related to angiogenesis in CRC. Li *et al*. identified through the miRNA target prediction program that Rh2 inhibits the growth and angiogenesis of CRC in SW620 and HCT-116 cells treated with Rh2. The mechanism is to increase the expression of miR-150-3p to restore the activity of the Wnt pathway, slow down cell proliferation/migration and colony formation, and reduce the generation of new blood vessels (Li et al., 2023). In addition, Rh2 also regulate the expression of miRNAs related to angiogenesis in A549 lung cancer cells (Chen et al., 2019).

The pH value of normal tissues is 7.2–7.5, and the pH value of the CRC tissue environment is generally 6.5–6.9. The acidic microenvironment of tumor cells is due to the tumor’s preference for aerobic glycolysis, enhanced pentose phosphate pathway, and hypoxia. High-speed aerobic glycolysis to maintain the required energy and carbon source is a metabolic marker of CRC cells, called the Warburg effect, leading to the accumulation of lactic acid (Zhong et al., 2022). Oncogenes such as EGFR (Maddalena et al., 2020; Gao et al., 2021), E2F1, c-myc (Jing et al., 2022) and Ras (Serna-Blasco et al., 2019) are important regulators of the Warburg effect. The mechanism of ginsenosides in regulating the acidic microenvironment may be related to the aforementioned hypoxia. Rh2 and Rd regulate the Warburg effect by inhibiting the activity of the EGFR pathway (Phi et al., 2018; 2019).

## 7 Ginsenosides show promising anti-cancer effects in clinical application

Current clinical research has confirmed that ginsenosides improve immune function and survival rate of tumor patients. Ginsenoside H pill (GH) is a new clinical adjuvant drug for cancer treatment. The main anti-cancer component of GH, Ginsenoside Rh2, reaches a steady state in the human body after oral administration of GH twice daily for five consecutive days (Wang et al., 2021). Aerobic exercise induces oxidative stress and DNA damage, reducing the incidence of CRC. Ginsenoside Rg1 protects the expression of skeletal muscle p16^*INK*4*a*^ protein induced by exercise in young men. It reverses the significantly increased p16^*INK*4*a*^ protein to close to baseline levels after 3 h (Wu et al., 2020). The combination of Ginsenoside Rg3 capsules and chemotherapy significantly improves the survival rate of postoperative patients with non-small cell lung cancer by improving immune function and inhibiting tumor angiogenesis (Lu et al., 2008). Ginsenoside Rg3 combined with transarterial chemoembolization (TACE) prolong the median overall survival of patients with advanced HCC. Rg3 also prolongs the time for the disease to progress to an incurable stage and reduces the adverse reactions related to TACE and hematological abnormalities (Zhou et al., 2016).

Ginsenoside is an immunomodulator used to treat CRC. Ginsenoside Rg3 treat CRC well (Tang et al., 2018). Its mechanism may be that Rg3 remodels the TME by inhibiting angiogenesis and promoting anti-tumor immunity (Zhao et al., 2022). Then, CRC downregulates its own immunogenicity by expressing high levels of PD-L1, which binds to the T cell receptor PD-1 to prevent the cytotoxic effect of T lymphocytes (Payandeh et al., 2020). Ginsenoside Rg1 inhibits the expression of PD-L1 through superoxide. Moreover, ginsenoside Rg1 inhibits the metastasis of CRC by interfering with the COX-2-Myo10 signaling axis and inhibiting filopodia production (Liu et al., 2023). Ginsenoside CK reverses the immunosuppressive TME. Ginsenoside CK significantly enhances the immune response of T cells while also increasing the ability of T cell receptors to recognize viral and tumor-associated antigens. Ginsenoside CK works synergistically with anti-PD-1, enhancing its anti-tumor efficacy (You et al., 2021). In addition, ginsenosides may treat CRC by reducing pro-inflammatory cytokines in the TME. It has been found that Rb1 significantly reduces the levels of TNF-α and IL-6 cytokines in mice with CRC cachexia models, alleviating symptoms caused by inflammation (Lu et al., 2020).

It is worth noting that CRC increases the psychological burden of patients, causing anxiety and depression (Cheng et al., 2022; Renna et al., 2022). Ginsenosides have the effect of anti-depression and improve the mood of cancer patients (Guan and Qi, 2023). The latest research found that ginsenoside Rh4 significantly inhibits the depressive-like behavior of depressed mouse models, alleviates neuronal damage and hypothalamic-pituitary-adrenal axis disorder, and inhibits hippocampal neuronal apoptosis and synaptic structural damage caused by excessive activation of microglia and astrocytes through the immune-inflammatory response and signaling molecule interaction pathway, thereby improving the depressive state (Shao et al., 2023). Ginsenoside Rh2 significantly reduce the depressive-like symptoms of mice induced by chronic unpredictable mild stress and downregulate the brain-derived neurotrophic factor (BDNF) signaling cascade and hippocampal neurogenesis, exerting an anti-depressant effect by positively regulating the BDNF-Tyrosine Kinase receptor B pathway (Shi et al., 2022). These studies suggest that ginsenoside Rh4 and Rh2 may be promising clinical drugs for treating anxiety and depression in CRC patients.

## 8 Conclusion and perspectives

Traditional Chinese medicine has a long history of treating CRC, with ginseng being one of the most important medicinal herbs. With advancements in medicine, our understanding of ginseng, especially its main active component—ginsenosides, has deepened. Currently, immunotherapy has become a breakthrough in colorectal cancer treatment. By searching electronic databases such as PubMed, Scopus, Web of Science and China National Knowledge Infrastructure, we obtained relevant literature on the treatment of CRC with ginsenosides and their immunomodulatory effects up to December 2023. The role of ginsenosides in the TME of CRC has been a research hotspot in recent years. We summarized the chemical structures, classifications, sources, and biosynthetic pathways of various ginsenosides. We documented the role of ginsenosides in promoting immune responses and inhibiting stromal cell activation within the colorectal TME. We summarized their effects on tumor cells and the TME, elucidating their mechanisms of action. Finally, we reviewed their clinical efficacy. This comprehensive review highlights the potential of ginsenosides as a promising immunomodulator in treating colorectal cancer.

According to the evidence summarized in this article, ginsenosides exhibit anticancer effects, including inducing cell cycle arrest and apoptosis, regulating autophagy, and reducing tumor invasiveness. Ginsenosides regulate the immune responses of myeloid and lymphoid cells within the TME. Ginsenoside Rh2, Rg3, and CK enhance the infiltration of CD8^+^ T cells into the transplantation tumor with MC38 cells and increase granzyme production by inhibiting the expression of the key checkpoint PD-L1 on tumor cells. Ginsenosides trigger the infiltration and cytotoxicity of CD4+/CD8+ T lymphocytes and NK cells, promote the conversion of M2 macrophages to M1 macrophages, enhance TAM secretion, and reduce the number of immunosuppressive Tregs. Additionally, ginsenosides exhibit anti-tumor effects, including inducing cell cycle arrest and apoptosis, regulating autophagy, and reducing tumor invasion. They also reverse hypoxia, acidity, and excessive angiogenesis in the TME, thereby slowing tumor progression. This evidence indicates that ginsenosides enhance immune responses within the colorectal tumor microenvironment, effectively converting “cold tumors” into “hot tumors.” This highlights their significant potential and feasibility in combination with immunotherapy or chemotherapy for the treatment of colorectal cancer. In clinical applications for CRC treatment, ginsenosides also exhibit good immunomodulatory effects, especially Rg1, Rg3, and Rh2. Ginsenosides alleviate depression and anxiety in CRC patients, enhancing their quality of life. With the successful market launch of Cardunelli in 2023, immunotherapy is expected to be a major breakthrough in CRC treatment. Ginsenosides, in synergy with immune checkpoint inhibitors, remodel the tumor immune microenvironment, inhibit tumor metastasis, and reduce adverse events associated with immunosuppressive drugs, greatly improving patients’ quality of life. Thus, ginsenosides represent a promising adjunct in the comprehensive management of CRC. Their integration into immunotherapy offers a hopeful pathway toward enhancing treatment outcomes.

Ginsenosides hold certain advantages and potential as an adjunctive and alternative therapy for CRC. Firstly, ginsenosides possess unique immunomodulatory and anti-tumor properties, targeting multiple sites and signaling pathways to exert multifaceted effects. They directly inhibit tumours and enhance immune responses within the TME. Secondly, ginsenosides exhibit favorable drug safety profiles. They maintain stability in the body and do not produce significant toxic side effects with long-term use, ensuring high safety for tumor patients. Thirdly, while exerting anti-tumor effects, ginsenosides also inhibit the pro-tumorigenic physical and chemical characteristics. They suppress excessive tumor angiogenesis and improve hypoxic and acidic microenvironments. However, there are limitations to the use of ginsenosides in CRC treatment. Firstly, although several studies based on cell and animal experiments indicate the potential efficacy of ginsenosides for CRC, high-quality and large-scale clinical studies are lacking to confirm their therapeutic effects. Secondly, the bioavailability of ginsenosides needs improvement. The gastrointestinal tract does not easily absorb most ginsenosides due to their low permeability through the intestinal epithelium. Future preparations of these herbal monomers could benefit from incorporating nanotechnology to prevent early release of active ingredients, thereby enhancing bioavailability and targeted therapeutic effects. Thirdly, the understanding of the molecular mechanisms by which ginsenosides regulate the tumor immune microenvironment is still superficial. More in-depth basic medical research is required to elucidate these mechanisms comprehensively. Therefore, developing ginsenoside-based therapies for CRC holds significant promise, warranting further investigation and optimization.

In summary, ginsenosides hold promising prospects as an adjunctive and alternative treatment for CRC. Traditional Chinese Medicine has a long history of clinical application. In patients with colorectal cancer undergoing chemotherapy and immunotherapy, ginsenosides exhibit synergistic anticancer effects and can reverse drug resistance in various cancer cells. This potential synergistic action may enhance the tolerance of chemotherapy and immunotherapy in clinical settings for colorectal cancer patients, thereby extending their overall survival. Current research and exploration of representative herbal monomers like ginsenosides can expand the therapeutic scope of Traditional Chinese Medicine and promote its international application. In drug development, the future combination of herbal monomers with nanotechnology, using nanocarrier materials to transport the monomers and prevent early drug release, will significantly enhance the bioavailability and clinical therapeutic effects of these compounds. We anticipate that ginsenosides will become a promising treatment option for CRC patients in the future.
